# Withdrawal Note of "Application of Cell-Based Assay Systems for the Early Screening ofHuman Drug Hepatotoxicity in the Discovery Phase of Drug Development"

**Published:** 2025-12-21

**Authors:** IJPR Archive

**Affiliations:** 1 School of Pharmacy, Shahid Beheshti University of Medical Sciences, Tehran, Iran

The mentioned above article was withdrawn by EIC descision.

